# Circulating Biomarkers Reflecting Destabilization Mechanisms of Coronary Artery Plaques: Are We Looking for the Impossible?

**DOI:** 10.3390/biom11060881

**Published:** 2021-06-14

**Authors:** Marko Kumric, Josip A. Borovac, Dinko Martinovic, Tina Ticinovic Kurir, Josko Bozic

**Affiliations:** 1 Department of Pathophysiology, University of Split School of Medicine, 21000 Split, Croatia; marko.kumric@mefst.hr (M.K.); jborovac@mefst.hr (J.A.B.); dinko.martinovic@mefst.hr (D.M.); tticinov@mefst.hr (T.T.K.); 2 Clinic for Cardiovascular Diseases, University Hospital of Split, 21000 Split, Croatia; 3 Department of Endocrinology, University Hospital of Split, 21000 Split, Croatia

**Keywords:** acute coronary syndrome, vulnerable plaque, atherosclerosis, biomarkers, myocardial infarction, plaque rupture, plaque erosion

## Abstract

Despite significant strides to mitigate the complications of acute coronary syndrome (ACS), this clinical entity still represents a major global health burden. It has so far been well-established that most of the plaques leading to ACS are not a result of gradual narrowing of the vessel lumen, but rather a result of sudden disruption of vulnerable atherosclerotic plaques. As most of the developed imaging modalities for vulnerable plaque detection are invasive, multiple biomarkers were proposed to identify their presence. Owing to the pivotal role of lipids and inflammation in the pathophysiology of atherosclerosis, most of the biomarkers originated from one of those processes, whereas recent advancements in molecular sciences shed light on the use of microRNAs. Yet, at present there are no clinically implemented biomarkers or any other method for that matter that could non-invasively, yet reliably, diagnose the vulnerable plaque. Hence, in this review we summarized the available knowledge regarding the pathophysiology of plaque instability, the current evidence on potential biomarkers associated with plaque destabilization and finally, we discussed if search for biomarkers could one day bring us to non-invasive, cost-effective, yet valid way of diagnosing the vulnerable, rupture-prone coronary artery plaques.

## 1. Introduction

Causing more than 1.8 million deaths a year worldwide, acute coronary syndrome (ACS) represents a major global health burden [1]. Adverse remodeling that occurs after myocardial infarction (MI) is a major contributor to the poor long-term outcomes in the ACS and in some patients this process is irreversible, thus stressing the importance of cardiovascular prevention as a crucial measure to avoid adverse cardiovascular events [2,3]. One of the plausible mechanisms to achieve this could be stabilization and/or regression of the existing plaque within the coronary vessels. The first step would be to adequately define vulnerable plaque in the clinical setting. It has so far been well-established that most of the plaques leading to ACS are not a result of gradual narrowing of the vessel lumen, but rather as a result of disruption of an atherosclerotic plaque which most commonly did not cause a hemodynamically significant stenosis of the coronary artery lumen [4]. These plaques have certain “ominous” characteristics that make them prone to rupture, and were thus named vulnerable plaques [4]. Notably, not all vulnerable plaques will rupture and result in cardiovascular events [5]. Even though coronary angiography is valuable and most used procedure in terms of diagnosis and treatment of the ACS, plaque pathobiology cannot be assessed using its classical modality, and it could thus result in wrongful discarding of most vulnerable plaques as harmless [6,7]. However, in the recent years, advancements in intravascular ultrasound (IVUS), high-resolution magnetic resonance imaging (MRI) studies positron emission tomography-computed tomography (PET-CT), and modern angiographic modalities with implemented optical coherent tomography (OCT) brought an in vivo insight into vulnerable plaques [8,9,10,11,12,13,14,15]. Intravascular OCT, a high-resolution (10–20 μm) light-based intravascular imaging technique, although bearing some noteworthy limitations, is currently the most relevant intracoronary imaging method as it can distinct vulnerable from stable plaque in a fairly reliable manner [16].

Hence, a search for different methods is warranted to appropriately recognize a vulnerable plaque in a timely manner. So far, most of the knowledge gathered regarding the plaque vulnerability and consequent rupture has been based on either animal models or patients with sudden cardiac deaths following the myocardial infarction [7,17,18]. Yet, pathological studies are very limited by their cross-sectional design and teleological nature, therefore, development of imaging tools which are capable of assessment of morphological characteristics in vivo were needed. Apart from various imaging modalities, multiple biomarkers were proposed to identify the presence of vulnerable plaques or plaques at different stages of remodeling. A logic behind measurement of biomarkers in blood that reflect the presence of vulnerable plaque is dual [19]. Certain molecules could arise as a consequence of leakage from unstable plaques, whereas others could merely indicate that a patient is susceptible to the development of a vulnerable plaque as those molecules are implicated in plaque destabilization. However, to the present day there are no clinically implemented biomarkers or any other methods for that matter that could non-invasively yet reliably diagnose the vulnerable plaque. Hence, in this review we present the available knowledge of pathophysiology behind vulnerable plaques, the current evidence on potential biomarkers reflecting plaque destabilization and finally we discuss if search for biomarkers could one day bring us to non-invasive, cost-effective, yet reliable way of diagnosing the rupture-prone coronary artery plaques.

## 2. Pathophysiology of Vulnerable Plaques

The detrimental cascade by which atherosclerosis develops in arteries is one of the most studied processes in humans [20]. However, not all of the aspects have been completely understood so far [21]. Changes in the constitutive properties of endothelial cells (ECs) on arterial-susceptible sites with increased endothelial shear stress (branch points and the outer wall of bifurcations) are the initial step of atherosclerotic plaque development [22]. Under the effects of subtle microenvironmental triggers from blood or interstitia, vascular ECs become dysfunctional and begin to synthesize an array of cytokines and chemokines whilst upregulating the expression of various cell-adhesion molecules, setting off a complex cascade that results in atherosclerotic plaque formation [23,24,25,26,27,28,29].

The crucial stage of the atherosclerotic process is creation of the unstable fibro-lipid plaque. However, it is now very important to acknowledge that, for reasons not completely understood, the vast majority of the atherosclerotic lesions, as many as 95%, will never result in an acute thrombo-occlusive vascular disease or any symptoms for that matter [30,31,32]. Recent analyses suggest that those vulnerable, rupture-prone plaques fall into two categories: The first being plaques with lipid-rich necrotic cores, thin fibrous caps and abundant in inflammatory cells, and the other being plaques characterized by excessive extracellular matrix and endothelial apoptosis [33]. Plaques with necrotic cores are primarily associated with ST-elevation myocardial infarction (STEMI) and their pathobiology has been well understood. The necrotic core of those plaques is a result of death of macrophages but also smooth muscle cells (SMCs) in the lesion combined with impairment in efferocytosis—i.e., poor phagocytic clearance of dead cells. The rupture itself is a consequence of fibrous cap thinning which arises from death of collagen-producing SMCs in the intima and upregulation of matrix-destroying proteases, whereas lipid-loaded core creates additional physical strain on the overlying fibrous cap making it even more susceptible to the rupture [34].

Unlike the former, matrix-rich lipid-poor plaques usually lack prominent macrophage collections and the main pathophysiologic mechanism that leads to ACS in these circumstances is superficial erosion, a poorly understood process not pertaining to pathogenetic mechanisms associated with plaque rupture [35,36,37,38]. As discussed by Libby et al., it seems that multiple processes predispose these plaques to superficial erosion, including flow disturbance, basement membrane breakdown, endothelial cell death, and detachment potentiated by innate immune activation mediated through pattern-recognition receptors, as well as endothelial–mesenchymal transition [39]. It is suggested that at least 50% of ACS arise as a consequence of plaque rupture, around 33% as a result of plaque erosion, whereas for the rest of ACS we have no clear proofs of either rupture or erosion [40,41,42,43].

Although currently there is no definitive evidence, it seems that the principal contributor to the plaque progression from virtually harmless stable into vulnerable rupture-prone plaque is an interplay between the aforementioned impaired efferocytosis and defective resolution of the inflammation in the advanced stages of atherosclerosis. The mechanism of defective efferocytosis in plaque progression is still largely misunderstood, yet it is quite clear that overwhelming apoptosis is not a contributor to the process [44]. Multiple authors favor the hypothesis that specific molecular-cellular processes involved in uptake or recognition of apoptotic cells by intralesional macrophages lead to impaired efferocytosis [45,46].

The concept of defective resolution implies failure of mechanisms that usually lead to repair of the inflammation-affected areas [47]. The resolution of inflammation is actually a very primal defense mechanism, that evolved concomitantly with inflammation in order to restore tissue function which has been deteriorated by inflammation’s detrimental effects. The resolution is mediated by multiple effector arms [48]: endogenous lipids called specialized pro-resolving mediators (SPMs), which include lipoxins, resolvins, protectins, and maresins; certain bioactive gases (nitric oxide, hydrogen sulphide, and carbon monoxide); anti-inflammatory proteins (IL-10, TGFβ, and annexin A); and resolving cells which include regulatory T cells (T_reg_) and resolving-type macrophages. In states of chronic inflammation, such as atherosclerosis, these effectors are impaired and lead to an amplification cycle of continuous tissue injury [49]. Multiple evidence suggests that atherosclerotic plaques have reduced levels of the above-noted effectors of resolution [49,50]. Accordingly, exogenous administration of these molecules has been shown to suppress the progression of mid-stage plaques to advanced plaques [51,52,53].

However, it still remains unclear as to what dampens the resolution in the setting of atherosclerotic plaque progression. There are three possible targets within the resolution framework that could be targeted: the production of resolution agents, excessive inactivation of those agents and finally, impaired response to those agents [54]. Multiple studies suggest that all of those mechanisms take part in impairment of resolution [51,52,55]. Interestingly, studies suggest that in phagocytes which accumulate apoptotic cells, i.e., when efferocytosis is preserved, production of SPMs is upregulated [56]. In addition, activation of the MerTK receptor, macrophage receptor involved in the process of efferocytosis, results in upregulation of SPMs production as well [57]. These observations highlight the mutual relationship between efferocytosis and resolution, two of the most viable mechanisms involved in progression of vulnerable atherosclerotic plaques. Finally, it is important to note that following the CV event, atherosclerotic plaque stabilizes over time, as demonstrated by Peeters et al. [58]. Plaque stabilization can be achieved by various mechanisms: by increasing of fibrous cap thickness, by reduction of inflammation in the fibrous cap and by reducing size of atheromatous core [59]. It is also noteworthy that plaques may be stabilized against thrombosis independent of changes in either plaque size or luminal obstruction [59].

## 3. Biomarkers of Vulnerable Plaques

**Figure 1:**
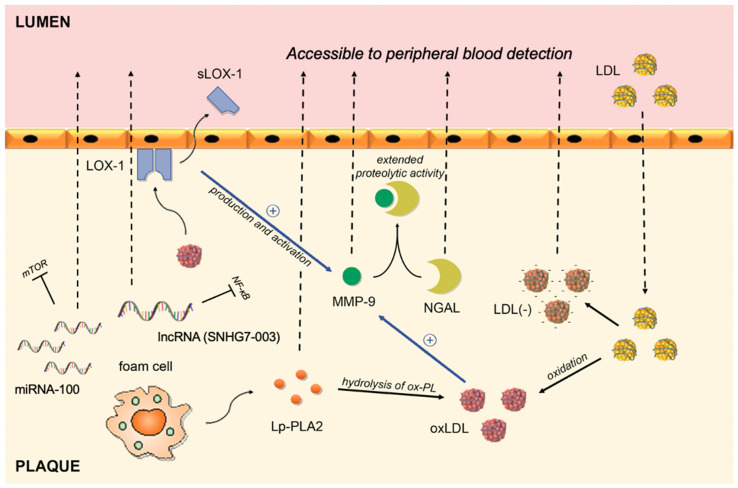
Circulating biomarkers of vulnerable plaques and their interplay in the intima of the coronary artery. LDL migrates from blood to plaque, where it goes through series of modifications. By binding to LOX-1, oxLDL activates and upregulates the production of MMP-9, whereas shedding of the LOX-1 receptor allows its peripheral blood detection. The complex between NGAL and MMP-9 results in extended proteolytic activity of MMP-9. Lp-PLA2 is responsible for hydrolysis of ox-PL on LDL particles and subsequent release of proinflammatory lipids. MMP-9: Matrix metalloproteinase-9; NGAL: Neutrophil gelatinase-associated lipocalin; sLOX-1: soluble part of the Lectin-like oxidized low-density lipoprotein receptor-1; Ox-LDL: oxidized low-density lipoprotein; Ox-PL: oxidized phospholipids; Lp-PLA2: lipoprotein-associated phospholipase A2; LDL(-): electronegative low-density lipoprotein; LDL: low-density lipoprotein; miRNA-100: microRNA-100; lncRNA: long non-coding RNA.

Since atherosclerosis is an everlasting field of research worldwide, there is a multitude of biomarkers whose potential to indicate a vulnerable plaque has been tested. Owing to the pivotal role of lipids and inflammation in the setting of atherosclerosis, most of the biomarkers originated from one of those processes. Conceptually, it is hypothesized that elevated levels of certain lipids will indicate increased susceptibility to the development of unstable atherosclerotic plaque owing to their role in the origin of plaque destabilization. On the other hand, certain molecules can migrate from vulnerable lesion back to circulation, thus creating an opportunity to serve as a biomarker (Figure 1). In addition, recent development of molecular science brought some novel technologies such as microRNA (miRNA).

### 3.1. Inflammation-Based Biomarkers

One of the most extensively studied biomarkers in this setting is high sensitivity C-reactive protein (hs-CRP), an established inflammatory biomarker used in everyday practice worldwide [60]. Aside from the well-known role as the most important acute-phase protein synthetized in the liver, increased CRP levels have been shown to be an independent risk factor for myocardial infarction, predicting CV events better than low-density lipoprotein (LDL) cholesterol [61,62,63]. Studies suggest that apart from the liver, CRP can be synthesized in plaques by macrophages or smooth muscle-like cells [64]. In line with this, Inoue et al. demonstrated CRP is released both from vulnerable coronary plaques and plaques damaged during percutaneous coronary intervention (PCI), whereas Norja et al. demonstrated that CRP immunoreactivity is associated with the progression of atherosclerotic plaque, especially with the vulnerable coronary plaques [65,66]. Apart from representing a biomarker of inflammation, studies suggest that CRP is an effector molecule able to induce a pro-atherothrombotic phenotype in endothelial cells and SMCs and is thus directly implicated in plaque pathophysiology [67]. Although CRP plasma levels undoubtedly reflect the vulnerability of the plaque, perhaps the biggest setback in its clinical implementation in this setting is its low specificity [68,69]. Virtually any inflammatory process in the human body can result in elevated CRP levels. However, CRP could be beneficial as a part of multiple biomarker prognostic score or if used in conjunction with imaging techniques.

Matrix metalloproteinase-9 (MMP-9) belongs to the family of zinc-binding proteolytic enzymes that are capable of degrading most of the extracellular matrix and that take part in all inflammatory processes in humans [70]. Expression of MMP-9 is markedly upregulated in human macrophages stimulated by oxidized low-density lipoprotein (ox-LDL), suggesting its contribution to matrix degradation in the atherosclerotic plaque and making it susceptible to rupture and/or vascular remodeling [71,72]. In line with this, studies on carotid plaques indicate that MMP-9 expression in carotid plaques is higher in vulnerable/symptomatic plaques in comparison to stable ones, whereas serum MMP-9 levels are significantly higher in patients with atheromatous plaques in contrast to patients with fibrous plaques [73,74,75,76]. Furthermore, multiple studies suggest that serum MMP-9 levels are elevated in patients with unstable plaques and Ferroni et al. suggest that MMP-9 serum levels might even provide an index of plaque activity in the setting of coronary artery disease (CAD) [77,78,79]. Notably, certain polymorphisms of MMP-9 confer a susceptibility risk for CAD. The most consistent evidence with respect to association of MMP-9 polymorphisms and CAD is that regarding the C1562T polymorphism. In a recent meta-analysis, Hassanzadeh-Makoui et al. demonstrated that MMP-9 (C1562T) polymorphism was associated with increased risk of CAD susceptibility in the overall analysis, but markedly in the Asian population [80]. The pathophysiological background of this association is the fact that this variant of MMP-9 decreases binding potential of the proteins involved in the inhibition of transcription to the DNA sequence, thus playing a role in orchestrating the transcription activity of MMP-9 [81]. Rather interestingly, this polymorphism exerts a protective role in a wide spectrum of diseases, such as diabetic nephropathy and anterior open bite [82,83]. In a large prospective trial (*n* = 1127) that included patients with CAD, the association between MMP-9 levels and the risk of fatal CV events showed a hazard ratio of 1.3, even after adjustment for confounders in terms of therapy and other clinical confounders whereas multivariate regression analysis by Ezhov et al. disclosed that MMP-9 is a strong independent predictor of plaque instability in stable CAD patients [84]. An important line of evidence with regard to MMP-9 implementation was brought by Wang et al. who showed that, in patients presenting with unstable angina, serum MMP-9 levels may discriminate patients who have unstable plaques from patients who do not have plaques, directly implicating a viable clinical usefulness of the MMP-9 [85]. However, further well-designed studies are needed to establish a putative role of this marker in everyday clinical setting.

A member of the lipocalin superfamily, Neutrophil gelatinase-associated lipocalin (NGAL) is an important regulator of the MMP-9 enzymatic activity and is thus implicated in progression of atherosclerosis [86]. NGAL creates a complex with MMP-9 which then inhibits degradation of MMP-9, consequently extending its proteolytic activity [86]. Circulating levels of both NGAL and MMP-9/NGAL complexes are significantly increased in asymptomatic patients with vulnerable carotid plaques, as demonstrated by Eilenberg et al. [87]. Multiple authors reported higher NGAL levels in patients with ACS in comparison to patients with stable CAD [88,89]. In addition, NGAL has been shown in several studies to correlate with poorer prognosis and to predict all-cause mortality and major adverse cardiac events (MACE) in patients with ACS [90,91,92]. However, implication of NGAL in a myriad of processes such as acute kidney injury, heart failure, and stable CAD, as well as inconsistence in data regarding prediction of clinical outcomes reduces its chance for establishment as a biomarker of plaque vulnerability [89,93,94].

Another molecule that requires attention is soluble part of the Lectin-like oxidized low-density lipoprotein receptor-1 (sLOX-1), produced by shedding of the LOX-1 [95]. Interaction between ox-LDL and its principal receptor, the LOX-1, appears to play a role in vascular dysfunction, including cells apoptosis and MMP production and activation, evoking the plaque rupture or erosion [96,97,98,99]. LOX-1 is abundantly present advanced in human atherosclerotic lesions. However, the main advantage of LOX-1 in terms of biomarker value is that its soluble part, the sLOX-1, is significantly elevated during the acute stage of ACS whilst not in general acute inflammatory diseases or stable CAD [100,101]. In fact, elevated sLOX-1 levels were detectable at an earlier stage after the onset of ACS in comparison to those of troponin-T (TnT), indicating that sLOX-1 reflects the atherosclerotic plaque vulnerability/rupture even before ischemic cardiac damage becomes clinically evident [100]. Furthermore, sLOX, but neither hsCRP nor hsTnT, can differentiate ACS with plaque rupture from those without, and ACS with thin-cap fibroatheroma from those without, as shown by OCT studies [102]. Additionally, the accuracy of the ACS diagnosis improved when sLOX-1 and hs-TnT were measured in combination [103].

### 3.2. Lipid-Based Biomarkers

It has so far been well established by large epidemiological studies that higher lipid levels correlate with the occurrence of major CV events [104,105,106]. Hence, since major CV events arise from vulnerable plaques, it is reasonable to infer that lipid status could indicate plaque vulnerability. Unlike in plasma, where LDL can be scarcely modified, in the atherosclerotic plaque, LDL is easily modified under the effect of free radicals and enzymatic activity [107]. More importantly, these modified LDLs gain inflammatory properties and become aggregated, impeding their return to the circulation [103,108]. However, a small portion of these reach plasma and it is therefore hypothesized that these could serve as biomarkers.

Ox-LDL is one of the key pathophysiologic contributors to the atherosclerotic plaque development and progression [109]. It has been demonstrated by multiple authors that amount of plaque ox-LDL correlates with plaque instability, especially in symptomatic carotid artery disease [110,111,112]. However, the translation of experimental evidence in humans with aimed at the demonstration of the association between ox-LDL plasma levels with CV events proved to be difficult as it resulted in contrasting findings [113,114,115,116]. This example highlights the challenging nature of plaque biomarker implementation in clinical practice, as although ox-LDL is a major participant in the in proinflammatory process associated with plaque rupture, its plasma levels do not seem to reliably reflect the vulnerability of the plaque.

Electronegative LDL (LDL(−)) is a modified fraction of the LDL that holds physical and chemical characteristics that differ from those of native LDL, such as increased lipoprotein-associated phospholipase A2 activity (Lp-PLA2), ceramide, clusterine, non-esterified fatty acid content, as well as increased aggregation level [117]. LDL(−) normally comprises around 3−5% of the total LDL plasma quantity, yet in certain CV pathologies this fraction rises [118]. A moderate rise of LDL(−) is commonly observed in patients with classical CV risk factors such as hypercholesterolemia, active smoking, diabetes, and metabolic syndrome [119,120,121,122]. Moreover, LDL(−) is an important mediator of atherogenesis which triggers the detrimental cascade by binding to LOX-1, the aforementioned inflammatory biomarker [123]. It has been recently shown that the most electronegative and the most atherogenic LDL fraction, the L5 fraction, is elevated in plasma of patients in STEMI [124]. In addition, L5 from STEMI patients can enhance platelet aggregation in vitro [125]. L5 levels have been also elevated in patients with ischemic stroke, and Shen et al. argue that L5 could thus serve as a marker of plaque vulnerability in those patients [125]. Even though this biomarker could be promising, a much larger body of well-design studies is needed to confirm it.

Lp-PLA2, originally named platelet-activating factor acetylhydrolase (PAF-AH), is an enzyme formed by macrophages and foam cells in atherosclerotic plaque where it is responsible for hydrolysis of oxidized phospholipids (ox-PL) on LDL particles and subsequent release of proinflammatory lipids [126]. It has been shown that stable atherosclerotic plaques contain only small amount of Lp-PLA2, unlike vulnerable plaques which are abundant in this molecule, markedly inside necrotic core [127]. Importantly, unlike CRP, which is elevated in most inflammatory processes, Lp-PLA2 represents a vascular-specific inflammatory marker and its plasma concentration is stable in terms of time, which largely increases the putative role of this molecule as a biomarker [128]. In addition, Lp-PLA2 level is independent of insulin resistance, unlike most of the other biomarkers [129]. In a study by Sarlon-Bartoli et al., authors demonstrated that patients with confirmed unstable carotid plaque, even if asymptomatic, have higher Lp-PLA2 plasma levels [130]. Similarly, Dong-Ling et al. demonstrated the same in patients with coronary artery plaques, highlighting that the specificity of serum Lp-PLA2 was stronger than that of hs-CRP in this setting [131]. Furthermore, multiple epidemiological studies and meta-analysis demonstrated that Lp-PLA2 is independently associated with the risk of CAD, but rather interestingly, not with atherosclerosis in abdominal aorta [132,133,134]. This discrepancy was further investigated by Fenning et al., which confirmed the differential role of Lp-PLA2 in the inflammatory cascade between different plaques [135]. The authors hypothesize that the observed discrepancy arises from differences in flow hemodynamics and embryologic origin of the vasculature. This discrepancy is very important from our point of view as it highlights the difficulty of using non-cardiac vasculature as a surrogate marker for detection and management of atherosclerotic plaques in coronary arteries. In summary, LpPLA2 seems to be a potentially beneficial adjunctive biomarker as it is both specific for vascular inflammation and, relatively speaking, for CAD.

### 3.3. Non-Coding RNAs

#### 3.3.1. MiRNAs

MiRNAs are short, single-stranded, non-coding RNA sequences that remain stable and are detectable in peripheral blood owing to inclusion in lipid or lipoprotein complexes [136]. The main function of miRNAs is post-transcriptional regulation of myriad of process in the human body [137]. The first reports on alterations in the expression of various miRNAs in human atherosclerotic plaques were brought by Cipollone et al. [138]. Among numerous muscle-enriched, vascular-enriched, myeloid cell-enriched miRNAs that were tested as markers of coronary plaque vulnerability by Soeki et al., only plasma miRNA-100 levels seem to indicate plaque vulnerability [139]. Authors have drawn this conclusion based on the findings that miRNA-100 levels were higher in coronary sinus blood samples in comparison to aortic blood samples and that transcoronary concentration gradients of circulating miRNA-100 positively correlated with percentage of plaque lipid volume and negatively with percentage of plaque fibrous volume. Soeki et al. argue that miRNA-100 is released into the coronary circulation from lipid-rich plaques as a form of compensatory reaction for plaque stabilization via suppressing the mammalian target of rapamycin signaling pathway. Another interesting miRNA in this setting is miRNA-21, miRNA whose role in atherosclerotic plaque progression has been well-established [140]. As demonstrated by Jin et al., vulnerable plaques and atherosclerotic lesions in apolipoprotein E (ApoE)-deficient mice exhibit lower miRNA-21 levels [141]. Moreover, mice-deficient in both ApoE and miRNA-21 were more prone to rupture, highlighting the implication of miRNA-21 in plaque instability. MiRNA-21 seems to enhance plaque stability by increasing SMC proliferation and consequent stabilization of the SMC-rich fibrous cap that shields the core of the plaque [141]. In a study by He et al., plasma levels of miRNA-21 correlated with several indicators of plaque instability, such as the size of the largest core area, thickness of the fibrous cap, and the lipid pool and the number of macrophages [142]. Several other studies demonstrated that miRNA-21 was upregulated in both patients with ACS and patients with ischemic stroke, as well as in stable CAD [143,144,145]. Apart from miRNA-100, more miRNAs have been recently associated with plaque burden (miR-126, miR-145, miR-155, and miR-29b). The miR-126 is particularly relevant, as its levels are associated with platelet function and can be modulated by antiplatelet treatment. As demonstrated by Carino et al., switching from dual antiplatelet treatment (acetylsalicylic acid +clopidogrel) to ticagrelor is associated with significant modulation in the circulating levels of certain miRNAs, paving the way for the use of circulating miRNAs as biomarkers of platelets activity in response to antiplatelet treatment [146].

However, miRNA technology currently bears some limits in terms of implementation in clinical practice [147]. The problem of normalizing plasma miRNAs levels is important, yet challenging. As Izawa et al. highlight, the expression profile of circulating miRNAs may change according to the current state of the patient, hence “housekeeping” miRNAs has not yet been established [148]. Consequently, a larger body of studies including large cohort of patients are needed to clarify whether circulating miRNAs could be useful as biomarker in this, but in other clinical settings as well.

#### 3.3.2. Long Non-Coding RNA (lncRNA)

LncRNAs were first identified as non-protein-coding RNAs with longer than 200 nucleotides in 1992 [149]. Since then, it has been discovered that by regulating gene expression and functions of other molecules through multiple approaches (epigenetic regulation, transcriptional regulation, post-transcriptional regulation, and many others), these non-coding RNAs have been implicated in a myriad of different pathophysiological processes in the human body [150]. We have been recently acquainted with the key role of lncRNA in inflammatory responses, thus implicating a potential link between lncRNA expression and atherosclerosis [151]. In fact, several studies have established this connection, paving a way for use of lncRNA in both diagnostic and treatment of CAD [152,153,154]. Plaque Enriched Long Noncoding RNA in Atherosclerotic Macrophage Regulation (PELATON) is a recently recognized lncRNA that has been upregulated in unstable atherosclerotic plaques [152]. Hung et al. demonstrated that key atherosclerotic processes of phagocytosis, ox-LDL uptake, and ROS production were all markedly affected by knockdown of PELATON, thus implicating that this lncRNA represents a potential target inhibition of plaque progression [152]. On the other hand, Pan et al. argue that lncRNA-SNHG7-003 has the potential to be a circulation lncRNA biomarker for evaluating plaque stability in patients with CAD, as this lncRNA was validated to be significantly downregulated in blood samples of patients with unstable plaques [153]. The pathophysiological background for this association lies in the ability of SNHG7-003 to inhibit LPS-induced activation of NF-κB pathway, thus regulating inflammatory response in human monocytes and macrophages [153]. Relevant biomarkers that have the potential to be used in the diagnosis of vulnerable plaques are presented in the Table 1.

## 4. Role of Invasive Imaging Methods in Plaque Pathobiology Assessment

Invasive coronary imaging methods, such as IVUS, have limited accuracy in identifying plaques likely to become culprit lesions [155,156,157]. In addition, as discussed by Bourantas et al., there are several other limitations that burden the use of those methods in vulnerable plaque detection [157]. As we previously mentioned, currently the most important invasive imaging modality is the intravascular OCT. OCT seems to be a powerful tool for discrimination of three types of unstable plaque morphologies underlying coronary thrombosis—such as plaque rupture, erosion, and calcified nodules—but also plaque healing [158]. All the pros and cons of OCT versus IVUS were discussed in a review by Su et al. [159]. The main advantage of OCT in comparison to IVUS is 10× higher resolution (15–20 µm vs. 150–200 µm) [160]. The discrepancy in resolution is also clinically relevant, as Lv et al. demonstrated that there are significant differences between IVUS and OCT plaque cap thickness measurements, the latter reflecting the plaque morphology more accurate [160]. Accurate plaque thickness is important as it can be used to predict plaque growth and vulnerability. On the other hand, OCT is characterized by lower tissue penetration than IVUS [160]. In addition, the development of hybrid intravascular imaging methods, which are currently undergoing preclinical evaluation, seem to enable more complete evaluation of the plaque pathophysiology [161]. In fact, there is an ongoing study, the ILUMIEN IV study, that is planned to be completed in 2022, and that will determine if OCT-guided stenting will provide better clinical outcomes than the angiographic guidance alone [159]. Additionally, near-infrared spectroscopy (NIRS) emerged as a viable method in this setting, as multiple studies have demonstrated that the detection of lipids in the walls of the coronary vessels identifies vulnerable patients at increased risk of new CV event [160]. Moreover, recent research regarding the combination of OCT and NIRS in plaque detection yielded promising results [161]. However, further large-scale trials are needed in order to support these notions.

## 5. Will There Be a Biomarker for Unstable Plaque in the Future?

Having regard to the search for clinically useful biomarker of coronary plaque vulnerability, the first issue that has to be properly addressed is: What do we intend to use it for? Do we merely want to determine the presence of a vulnerable plaque in coronary artery or do we want to establish exactly what chance of rupture and consequent CV event does the certain plaque have? The former seems to be more achievable than the latter, since in theory there should only be one threshold according which we would, with certain sensitivity and specificity, determine the presence or absence of a vulnerable plaque. Accordingly, such biomarker would be an appropriate screening method for invasive strategies such as OCT or IVUS, based on which we would determine further therapeutic strategies. On the other hand, risk stratification by rupture liability would require multiple thresholds, each with their respective level of sensitivity and specificity.

An ideal biomarker for vulnerable plaque would have a lot of milestones to achieve. Firstly, as for any biomarker, sensitivity and specificity should be sufficient. In this setting, given the potential threat that vulnerable plaque represents for an individual, we believe that search for biomarker should be focused more on sensitivity, whereas lack of specificity could be overcome with additional invasive methods as we discussed. Additionally, with regard to specificity, it is important to acknowledge that atherosclerotic plaque formation occurs throughout all of the large arteries. Hence, the elevation of biomarker plasma level would reflect the presence of plaque in any of the arteries in the human body. However, this characteristic could be also beneficial as presence of vulnerable plaque in some of the arteries that are easily accessible for ultrasound evaluation, such as carotid or femoral artery, could indicate presence of vulnerable plaque in coronary artery which is much less accessible to non-invasive methods. This is also supported by multiple observational studies which demonstrated that carotid intima-media thickness and carotid plaque presence are predictor of future coronary events [162,163]. Yet, the presented example of LpPLA2 teaches us that we must also bear in mind the site-specific characteristics of plaques. Furthermore, biomarker should be tested in large populational studies to confirm benefits in terms of outcomes, especially in comparison to the available clinical tools. Finally, the cost-effectiveness and accessibility is a major contributor to biomarker implementation, especially in poverty-stricken areas. So far, no biomarker of plaque vulnerability seems to fully attain these requirements.

Regarding the limitations of serum biomarkers and imaging studies for vulnerable plaque detection, Verhoeven et al. came up with a clever concept later referred to as the Athero-Express study [164]. In this study, atherosclerotic plaques were harvested during either carotid or femoral endarterectomy and subjected to histological analysis and protein isolation in order to evaluate inflammatory interleukins, MMPs and other inflammatory markers. Furthermore, patients were then followed after endarterectomy for three years and endpoints were registered. The main goal of the Athero-Express study was to establish a relation between plaque properties and CV events during follow up. It is important to acknowledge that the Athero-Express study itself did not challenge the definition of the vulnerable plaque, but used its concept in favor of outcome improvement for vascular patients. A decade of experience with the Athero-Express study brought us several important findings with respect to vulnerable plaques [165]. Firstly, it confirmed the concept that local plaque composition can predict systemic CV outcomes [166,167]. This conclusion was extrapolated from the fact that plaque hemorrhage and neovascularization, but not other histological parameters (size of the lipid core, macrophage infiltration, collagen, and SMC infiltration), were associated with secondary CV events during clinical follow-up [167]. Importantly, this finding highlights the importance of plaque hemorrhage and vascularization in pathophysiology of plaque progression, and thus addresses the need for focus on these characteristics in search for vulnerable plaques. Fortunately, intra-plaque hemorrhage can be visualized using contrast enhanced MRI, an expensive yet accessible and non-invasive diagnostic method [168]. Secondly, Athero-Express showed that although over-expression of the aforementioned MMP-9 has been implied as a major factor contributing to degradation of the fibrous cap and its plasma levels were higher in symptomatic patients, MMP-9 plaque expressions did not show any association with clinical outcomes [169,170]. Thirdly, it has been shown that stages of intraplaque hemorrhage are associated with different plaque phenotypes [171,172,173]. Finally, results from the Athero-Express study address the importance of the role of plaque composition in clinical decision making in CV diseases [174].

Although an abundance of studies implies the utility of various biomarkers in this setting, we are still far away from clinical implementation of either of those. Among all the biomarkers we presented, we believe that either sLOX-1 alone or in combination with MMP-9 are the only markers that have some chance of becoming markers of plaque vulnerability in routine clinical practice. Namely, there are some crucial characteristics that make them viable for this matter. The main advantage of sLOX-1 is its ability to discern vascular inflammation from other general inflammatory states. Furthermore, lack of correlation with TnT implies that sLOX-1 does not reflect cardiac tissue injury and very early peak of sLOX-1 serum levels in the setting of ACS (maybe even before the onset of ACS) suggests the correlation with plaque vulnerability. Finally, both MMP-9 and sLOX-1 were able to differentiate patients who had vulnerable plaques from those who did not, among group of patients presenting with unstable angina and ACS, respectively [85]. In the future we may use them as a part of stepwise approach, similar to the one proposed in the BioImage study [175]. Risk models that would rely on these or some other biomarkers could initially be used to identify intermediate-risk/high-risk patients who would benefit the most from more invasive modalities by which we would really establish the presence of the vulnerable plaque and decide on therapeutic interventions. Here is where we face the real problem. Namely, as we discussed, invasive imaging methods have limited accuracy in identifying these plaques, thus additionally impeding the process of plaque vulnerability recognition in a clinical setting.

Notably, development of artificial intelligence could in the future predict changes in plaque vulnerability [16,176]. In a feasibility study by Guo et al., the authors demonstrated that machine learning methods could be used to accurately predict changes in plaque vulnerability using morphological and biomechanical factors from multi-modality image-based 3D fluid–structure interaction coronary model [176]. Interestingly, in a recent study by Pan et al., author used multi-physical model for identification of the spatial-temporal dynamics and progression in coronary atheroma microenvironment [177]. Using the multi-physical model, mathematical model based on previous work from the same group, authors integrated the dynamic microenvironmental indicators with the classical risk factors in order to differentiate plaque progression to either stable or unstable plaques [178,179].

In summary, as reliable imaging modalities for vulnerable plaque detection are yet to be fully developed, we are still miles away from implementation of biomarkers in this setting. Simply, there would be no purpose of measurement of those biomarkers if it will not direct our clinical decision-making. However, given the potential benefits, once these issues are resolved, we must not undermine the importance of atherosclerotic plaque biomarker research.

## 6. Conclusions

Even though a myriad of biomarkers which should identify vulnerable plaque was developed in the recent years, the lack of reliable referent method for description of plaque vulnerability prevented us from implementation of any of those. Among the presented biomarkers, we believe that sLOX-1, alone or in combination with MMP-9, have the most suitable characteristics for becoming putative biomarkers in this setting in years to come. In addition, a major contributor for resolving the aforementioned issues could be machine learning methods, as preliminary results suggest that these could be used to accurately predict changes in plaque vulnerability. Finally, despite significant setbacks, having regard to the burden of the ACS, one must not undermine the importance of atherosclerotic plaque biomarker research.

## Figures and Tables

**Table 1:** Biomarkers in diagnostic approach to vulnerable plaque.

Biomarker	Pathophysiological Pathway	Supporting Evidence
hs-CRP	An acute phase protein that, apart from liver, can be synthesized in plaques by macrophages or smooth muscle-like cells	Inoue et al. [65] Norja et al. [66]
MMP-9	A proteolytic enzyme capable of degrading the extracellular matrix; upregulated in human macrophages stimulated by ox-LDL	Ezhov et al. [84] Wang et al. [85]
NGAL	Creates a complex with MMP-9 that inhibits degradation of MMP-9, thus extending its proteolytic activity	Eilenberg et al. [87]
sLOX-1	A soluble form of LOX-1 receptor; interaction between ox-LDL (ligand) and LOX-1 (receptor) plays a role in vascular dysfunction	Hayashida et al. [100] Ueda et al. [101] Kobayashi et al. [102]
Ox-LDL	An oxidized fraction of the LDL; major participant in the proinflammatory processes associated with plaque rupture	Wang et al. [110] Wang et al. [111] Sigala et al. [112]
Electronegative LDL	A modified fraction of the LDL; physical and chemical characteristics differ from native LDL (increased Lp-PLA2 activity, ceramide, clusterine, non-esterified fatty acid content, increased aggregation level)	Lu et al. [123] Yang et al. [124]
Lp-PLA2	An enzyme formed by macrophages and foam cells; hydrolyses oxidized phospholipids on LDL particles and subsequently releases proinflammatory lipids	Sarlon-Bartoli et al. [130] Dong-Ling et al. [131] Fenning et al. [135]
MicroRNA-100	Post-transcriptional regulation	Soeki et al. [139]
MicroRNA-21	Post-transcriptional regulation	Jin et al. [141] He et al. [142]

hs-CRP: high sensitivity C-reactive protein; MMP-9: Matrix metalloproteinase-9; NGAL: Neutrophil gelatinase-associated lipocalin; sLOX-1: soluble part of the Lectin-like oxidized low-density lipoprotein receptor-1; Electronegative LDL: electronegative low-density lipoprotein; Ox-LDL: oxidized low-density lipoprotein; Lp-PLA2: lipoprotein-associated phospholipase A2.

## Data Availability

Not applicable.
